# Video Game Intervention for Sexual Risk Reduction in Minority Adolescents: Randomized Controlled Trial

**DOI:** 10.2196/jmir.8148

**Published:** 2017-09-18

**Authors:** Lynn E Fiellin, Kimberly D Hieftje, Tyra M Pendergrass, Tassos C Kyriakides, Lindsay R Duncan, James D Dziura, Benjamin G Sawyer, Linda Mayes, Cindy A Crusto, Brian WC Forsyth, David A Fiellin

**Affiliations:** ^1^ play2PREVENT Lab Yale School of Medicine, Yale University New Haven, CT United States; ^2^ Yale Child Study Center New Haven, CT United States; ^3^ Center for Interdisciplinary Research on AIDS Yale School of Public Health, Yale University New Haven, CT United States; ^4^ Yale School of Medicine, Yale University New Haven, CT United States; ^5^ Yale School of Public Health, Yale University New Haven, CT United States; ^6^ McGill University Montreal, QC Canada; ^7^ Digitalmill Freeport, ME United States; ^8^ University of Pretoria Department of Psychology Pretoria South Africa

**Keywords:** adolescent, videogame, intervention, randomized controlled trial, human immunodeficiency virus, risk reduction, primary prevention

## Abstract

**Background:**

Human immunodeficiency virus (HIV) disproportionately impacts minority youth. Interventions to decrease HIV sexual risk are needed.

**Objective:**

We hypothesized that an engaging theory-based digital health intervention in the form of an interactive video game would improve sexual health outcomes in adolescents.

**Methods:**

Participants aged 11 to 14 years from 12 community afterschool, school, and summer programs were randomized 1:1 to play up to 16 hours of an experimental video game or control video games over 6 weeks. Assessments were conducted at 6 weeks and at 3, 6, and 12 months. Primary outcome was delay of initiation of vaginal/anal intercourse. Secondary outcomes included sexual health attitudes, knowledge, and intentions. We examined outcomes by gender and age.

**Results:**

A total of 333 participants were randomized to play the intervention (n=166) or control games (n=167): 295 (88.6%) were racial/ethnic minorities, 177 (53.2%) were boys, and the mean age was 12.9 (1.1) years. At 12 months, for the 258 (84.6%) participants with available data, 94.6% (122/129) in the intervention group versus 95.4% (123/129) in the control group delayed initiation of intercourse (relative risk=0.99, 95% CI 0.94-1.05, *P*=.77). Over 12 months, the intervention group demonstrated improved sexual health attitudes overall compared to the control group (least squares means [LS means] difference 0.37, 95% CI 0.01-0.72, *P*=.04). This improvement was observed in boys (LS means difference 0.67, *P*=.008), but not girls (LS means difference 0.06, *P*=.81), and in younger (LS means difference 0.71, *P*=.005), but not older participants (LS means difference 0.03, *P*=.92). The intervention group also demonstrated increased sexual health knowledge overall (LS means difference 1.13, 95% CI 0.64-1.61, *P*<.001), in girls (LS means difference 1.16, *P*=.001), boys (LS means difference 1.10, *P*=.001), younger (LS means difference 1.18, *P*=.001), and older (LS means difference=1.08, *P*=.002) participants. There were no differences in intentions to delay the initiation of intercourse between the two groups (LS means difference 0.10, *P*=.56).

**Conclusions:**

An interactive video game intervention improves sexual health attitudes and knowledge in minority adolescents for at least 12 months.

**Trial Registration:**

Clinicaltrials.gov NCT01666496; https://clinicaltrials.gov/ct2/show/NCT01666496 (Archived by WebCite at http://www.webcitation.org/6syumc9C0).

## Introduction

### Background

Human immunodeficiency virus (HIV) disease and sexually transmitted infections (STIs) significantly impact young people, with racial/ethnic minority youth disproportionately affected. Nearly 10,000 youth were diagnosed with HIV in 2014 in the United States, accounting for 22% of new infections [1]. There is a considerable range in both the quantity and quality of education around sexual health, HIV, and acquired immune deficiency syndrome (AIDS) adolescents receive. Only 22 states and the District of Columbia require that public schools teach sex education. Nineteen states require that if sex education is taught, it must be factually accurate [2]. Another 12 states require HIV/AIDS education. Although there are effective HIV and STI prevention programs, challenges in their implementation and fidelity exist. Service providers may lack access to programs [3], selectively implement program components [4], or never implement programs at all [5]. Barriers to implementation include access to adequately trained providers, resource constraints, fidelity, and challenges of adapting an intervention from one population to another [6-8]. To address these issues, digital health interventions have demonstrated efficacy at influencing sexual health [9,10], featuring adaptable content for broader reach, with greater fidelity, at a potentially lower cost [11].

Serious games, defined as video games for a primary purpose beyond pure entertainment [12], offer unique benefits in targeting health promotion and risk prevention [13]. They have efficacy [14] in areas ranging from depression to asthma to cancer [15,16-19]. They also have considerable reach, with 97% of adolescents, including all racial/ethnic groups, playing video games with 50% playing for at least 1 hour or more per day [20]. In a study assessing the impact of digital technologies on schools, more than 333,000 students in grades 6 to 12 reported wanting to use digital games for learning in school and reported games increased their engagement [21]. Active participation through simulated role-playing [22,23] in video games allows individuals to acquire knowledge and practice behavioral skills in a highly accessible, portable, and engaging way [16,24].

### Aim of This Study

Our objective was to test the efficacy of a digital health intervention in the form of a theory-driven interactive video game intervention compared to a set of control games on sexual risk behaviors, knowledge, attitudes, and intentions in a population of racial/ethnic minority adolescents. We hypothesized that a highly engaging theory-based video game intervention would have a greater impact on our outcomes of interest than a set of control games.

## Methods

### Study Design

We conducted a randomized controlled trial in 12 urban community-based settings consisting of seven school-based afterschool programs, four independent afterschool programs, and one summer camp. A description of the development of the PlayForward: Elm City Stories (PlayForward) intervention and the trial design have been published [25,26]. The Yale School of Medicine Human Investigation Committee approved the research.

### Participants

Participant eligibility included ages 11 to 14 years, speaks English, able to provide assent and parental/legal guardian consent, and willing to play video games for up to 75 minutes twice weekly for 6 weeks. Although the primary outcome of this study was delay of initiation of sexual intercourse, we enrolled participants who had already initiated intercourse because we did not want potential participants to provide incorrect information regarding their sexual activities to gain or avoid access to the study. These participants (n=6) were not included in these analyses. Participants were provided with an age-appropriate study description and parents/legal guardians were informed that the study focused on promoting healthy behaviors and reducing risk in adolescents. If an adolescent or parent/guardian did not wish to participate, they were not included in the study. All data were collected from 2013 to 2015 and analyzed in 2016.

### Randomization

Enrolled participants were randomized in an unmasked fashion to the PlayForward group or to a set of 12 attention-time control video games with all participants playing the games on iPad tablets. A single randomization scheme was generated and written in TrialDB, a customizable Web-based clinical trials database system [27]. After obtaining written youth assent and parental/legal guardian informed consent, eligible participants were assigned to play PlayForward or attention/control games in a 1:1 ratio using a computerized single randomization scheme. Randomization was stratified by gender and age group (11-12 years and 13-14 years), both predictors of the outcomes of interest [28,29]. Notably, gender differences have been identified as being important not only in terms of the trajectory of sexual behaviors in boys and girls during adolescence but also in terms of their response to interventions targeting sexual risk [29]. Randomization was under the control of an investigator who was not involved with eligibility assessment. Study personnel accessed the computerized randomization system, retrieved the assignment, and notified participants of their group assignment.

### Study Conditions

Participants played PlayForward or a set of control games on the iPad. PlayForward is a two-dimensional, role-playing adventure video game [25,30-33] developed using a theoretical foundation [32,34-37] about how individual choices made within the social environment of life impact both short-term and long-term goals (Multimedia Appendix 1). These theories primarily focus on the individual’s decision making, yet account for the fact that the decisions are made within a certain social context. Delivered as an interactive video game, the intervention allows for the player to see how their individual decisions are influenced by their social surroundings. The game consists of approximately 16 hours of gameplay. The player’s goal is to acquire and practice skills to reduce risk behaviors and gain knowledge and healthier attitudes and intentions with the ultimate goal of HIV prevention. The game involves an interactive world where the player creates an Aspirational Avatar [32] and “travels” through life, facing challenges and making decisions in the context of a series of narratives depicting common social situations whose outcomes bring different risks and benefits (Multimedia Appendix 2). The game focuses on sexual health and risk and a range of risky behaviors including substance use, academic dishonesty, and unsafe driving. Five skill-based interactive “mini-games” (Multimedia Appendices 3 and 4) are combined with 12 story-based “challenges” comprising the overarching narratives.

The attention/time control games consisted of 12 video games such as Angry Birds, Dragonbox, and Subway Surfer. They were devoid of content relevant to our study goals and mirrored the number of sessions and length of gameplay in the experimental group [38]. We considered a conventional prevention education control condition; however, there was no “gold standard” for teaching sexual education or HIV prevention in US schools [2], therefore using HIV prevention/education materials as the control was not consistent with current “treatment as usual.” We opted for an attention/time control because our primary goal was to determine the efficacy of the video game intervention.

Participants played their assigned game(s) for two sessions per week, approximately 1 hour per session, for 6 weeks on-site at their program [26]. Similarly, all assessments were conducted at the participant’s program and included data collected at baseline, 6 weeks (immediately after completion of gameplay), and at 3, 6, and 12 months.

The PlayForward game software records in-game data assessing intervention exposure and fidelity [39]. In-game data documents player’s actions, time spent on each action, and overall performance providing a measure of exposure to each intervention component. Periods of inactivity (eg, player is away from the device) can be identified from activity logging data timestamps. Research personnel observed and documented both the PlayForward and the control group participants’ duration of gameplay.

### Outcome Measures

The primary outcome was delay of initiation of sexual intercourse (defined as initiation of vaginal or anal intercourse) at 12 months post-baseline [40-42]. On June 1, 2015, before any data analyses, the investigators and the Data and Safety Monitoring Board (DSMB) clarified that the primary outcome would be assessed at 12 months postrandomization. Secondary outcomes were sexual attitudes, sexual health knowledge, and sexual intentions. Items assessing the secondary outcomes were compiled from standard instruments and assessed for their internal consistency using Cronbach alpha: delay of the initiation of intercourse [41], sexual attitudes [43] (3 items, maximum score=12, Cronbach alpha=.90), sexual health knowledge [44,45] (15 items, maximum score=15, α=.68), and intentions to delay intercourse [43] (four items, maximum score=16, Cronbach alpha=.84). Although the sexual health knowledge measure (which was piloted before the trial) had a slightly lower level of reliability than is considered acceptable (Cronbach alpha=.7), the items in this measure reflected content that was specifically built into the game, representing very good content validity, which is an important component of a knowledge test in terms of demonstrating that the items within the test are closely related to one another. In addition, although the Cronbach alpha was slightly lower than the .7 value, the result was not borderline, but considerable. Additional assessment data were collected but were not the focus of this paper [26]. Outcomes were assessed at 6 weeks and at 3, 6, and 12 months after randomization. Data were collected face-to-face by research staff and entered into a Web-based database. As described elsewhere [26], we collected data from participants individually; for assessments including sensitive data, participants filled out responses after questions were read to them. Measures were taken to ensure participants felt their responses were being kept confidential.

### Statistical Analysis

Power calculations were based on data from published studies [46,47] and systematic reviews [48-50] evaluating youth HIV prevention interventions. These studies suggested a small to moderate effect size of interventions on delaying/preventing initiation of sexual intercourse. National data indicated that approximately 7% of youth reported vaginal or anal intercourse before age 13 years [51] and the final sample size accounted for this figure. A sample size of 330 with 165 in each study condition, was estimated to detect a 15% difference in the proportion of participants achieving the primary outcome of delay of initiation of sexual intercourse (90% PlayForward vs 75% control groups), providing a power of 80% or greater to detect significant (two-tail alpha=.05) differences of this magnitude [52]. This sample size also afforded adequate power (>90%) to detect small to moderate effects on secondary outcome measures. NQuery version 4.0 was used to estimate sample size. This sample size accounted for losses in primary outcome assessment and for enrollment of participants who at baseline had already initiated sexual intercourse.

The primary comparison evaluated the effect of PlayForward compared with the control video games on the delay of initiation of sexual intercourse measured at 12 months postrandomization and constructed as a binary outcome (delayed beyond 12 months/initiated before 12 months). The primary analysis was carried out as an intent-to-treat analysis and adjusted for gender and age (the randomization stratification variables). Sensitivity analyses were carried out with missing responses being assigned both as delay and not delay of initiation of intercourse.

Differences in scores in the secondary outcomes were compared between the two groups at the time points using longitudinal mixed-effects models. Changes in secondary outcome measures since baseline were assessed in repeated measures models (with unstructured covariance), with the assigned baseline values, study group, gender, age, study time point, and study group*time interactions used as covariates. Least squares (LS) means and standard error were plotted for each secondary outcome at each time point. Statistical analyses were done using SAS version 9.4 (SAS Institute Inc, Cary, NC, USA). All protocols were reviewed at intervals by a DSMB.

## Results

### Description of Study Sample

**Figure 1:**
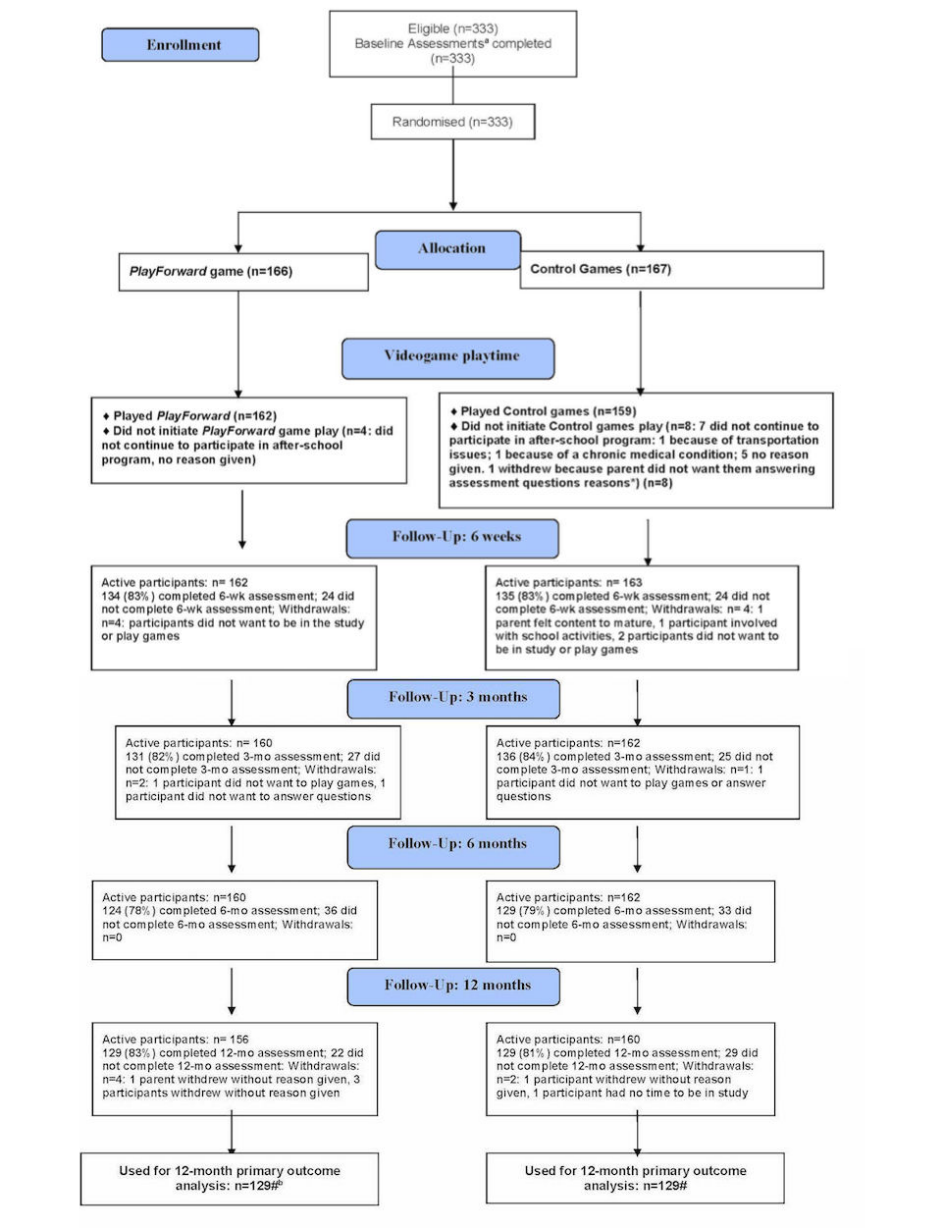
Enrollment and follow-up flow diagram for videogame intervention trial for sexual risk reduction. Note: Assessment refers to primary outcome assessment (delay of initiation of sexual intercourse);% is of active participants. Participants who did not initiate game play were still considered active and assessed for study outcomes. A total of six participants (four in PlayForward; two in control) who had initiated sexual intercourse (per primary outcome definition) at baseline were removed from the analysis of primary outcome.

A total of 333 participants were recruited and enrolled into the study between February 26, 2013 and May 16, 2014; 166 were assigned to the PlayForward intervention and 167 were assigned to the control condition (Figure 1). A total of 166 participants were assigned to PlayForward (162/166, 97.6% initiated gameplay) and 167 to the control games (159/167, 95.2% initiated gameplay). Reasons for not initiating gameplay included participants’ inability to participate in the afterschool program due to transportation or medical issues. The PlayForward group played a median of 10.1 hours (interquartile range (IQR) 3.9) over 10 sessions for a median of 60.4 minutes/session (IQR 11.6). The control group played a median of 10.1 hours (IQR 3.9) over 10 sessions for a median of 61.4 minutes/session (IQR 10.9). Eighteen participants withdrew leaving 315 participants in active follow-up. A total of 269 (82.7%) completed the 6-week assessments, 267 (82.9%) completed the 3-month assessments, 253 (78.6%) completed the 6-month assessments, and 258 (81.6%) completed 12-month assessments. No demographic or clinical differences by intervention and control conditions were observed at baseline (Table 1).

### Outcomes

The primary outcome was delay of initiation of sexual intercourse. Six participants (PlayForward: n=4; control: n=2) who had engaged in intercourse before baseline were removed from the primary analysis because they had already reached the primary outcome (Table 2).

Overall, the rates of initiating sexual intercourse were low in both groups. There were no differences in rates of delaying initiation of intercourse at each time point in the PlayForward versus the control groups. At 12 months, for the 258 of 304 (84.9%) participants from whom data were available, 122 of 129 (94.6%, 95% CI 89.1%-97.8%) in the PlayForward group versus 123 of 129 (95.4%, 95% CI 90.2%-98.3%) in the control group had delayed the initiation of intercourse (relative risk=0.99, 95% CI 0.94-1.05, *P*=.77). Sensitivity analysis suggested no difference in these proportions (missing=delay, *P*=.90; missing=no delay, *P*=.78).

**Figure 2:**
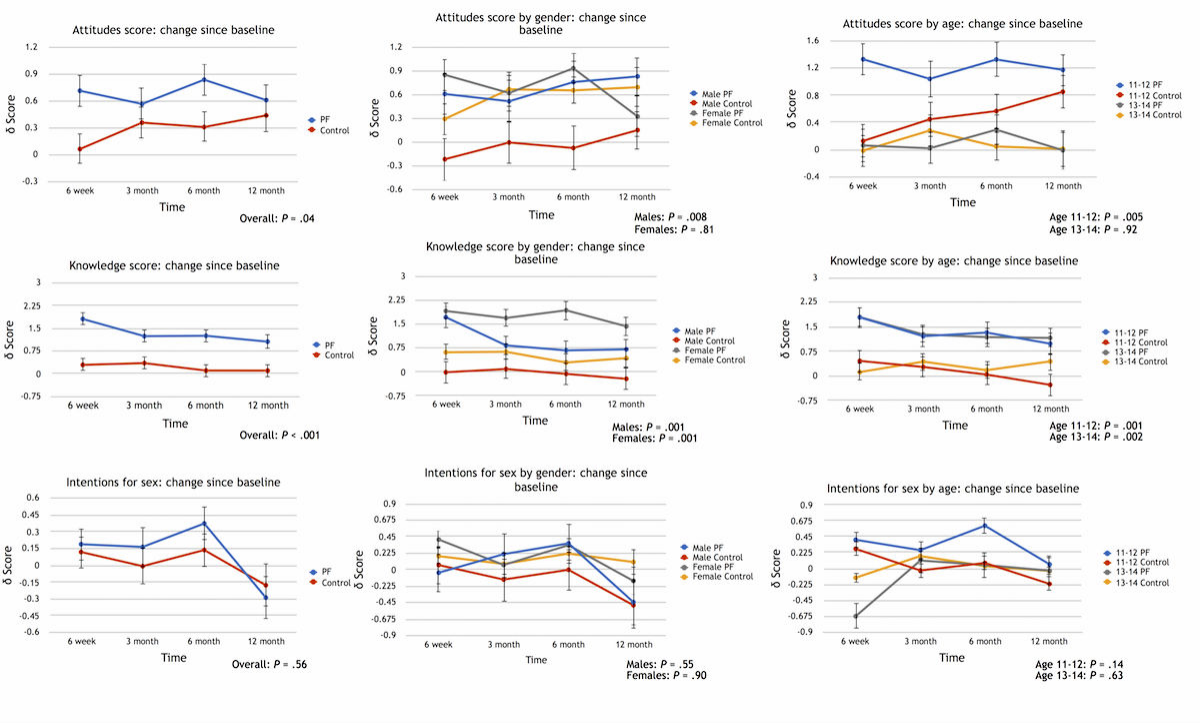
Changes in attitudes, knowledge, and intentions by study condition for total group by gender and by age. PF: PlayForward.

Over the 12-month follow-up period, the PlayForward group demonstrated an improvement in attitudes about sexual health compared to the control group (LS means difference 0.37, 95% CI 0.01-0.72, *P*=.03) (Figure 2). Treatment*age group and treatment*gender interactions were not significant (*P*=.06 and *P*=.09, respectively). The improvement in attitudes was not seen in girls (LS means difference 0.06, 95% CI –0.46 to 0.58, *P*=.81), but was seen in boys (LS means difference 0.67, 95% CI 0.18-1.16, *P*=.008). In addition, this improvement was only seen in the younger group of participants (age 11-12 years: LS means difference 0.71, 95% CI 0.21-1.20, *P*=.005) and not the older group of participants (age 13-14 years: LS means difference 0.03, 95% CI –0.49 to 0.54, *P*=.92).

Over the 12-month follow-up period, the PlayForward group demonstrated an increase in sexual health knowledge compared to the control group (LS means difference 1.13, 95% CI 0.64-1.61, *P*<.001) (Figure 2). Both girls and boys in the PlayForward group demonstrated an increase in sexual health knowledge compared to the control group (girls: overall LS means difference 1.16, 95% CI 0.46-1.86, *P*=.001; boys: LS means difference 1.10, 95% CI 0.43-1.77, *P*=.001). Both younger (age 11-12 years) and older (age 13-14 years) participants in the PlayForward group demonstrated an increase in sexual health knowledge compared with the control group (younger: LS means difference 1.18, 95% CI 0.50-1.85, *P*=.001; older: LS means difference 1.08, 95% CI 0.39-1.78, *P*=.002).

Over the 12-month follow-up period, there were no differences in intentions to delay the initiation of intercourse between the two groups (LS means difference 0.10, 95% CI –0.23 to 0.43, *P*=.56) (Figure 2). Similarly, there were no differences noted over the 12-month follow-up period by gender or by age group.

**Table 1:** Baseline demographic and clinical characteristics of participants.

Characteristics		Control (n=167)	PlayForward (n=166)	Total (N=333)
**Gender, n (%)**				
	Male	89 (53.3)	88 (53.0)	177 (53.2)
	Female	78 (46.7)	78 (47.0)	156 (46.8)
Age (years), mean (SD)		12.9 (1.1)	12.9 (1.1)	12.9 (1.1)
**Age group (years), n (%)**				
	11	41 (24.6)	42 (25.3)	83 (24.9)
	12	45 (26.9)	45 (27.1)	90 (27.0)
	13	45 (26.9)	45 (27.1)	90 (27.0)
	14	36 (21.6)	34 (20.5)	70 (21.0)
**Race, n (%)**				
	White	14 (8.6)	17 (10.4)	31 (9.5)
	Black	65 (40.1)	70 (42.7)	135 (41.4)
	Other	80 (49.4)	76 (46.3)	156 (47.9)
	Unknown	3 (1.9)	1 (.6)	4 (1.2)
**Ethnicity, n (%)**				
	Hispanic	87 (55.1)	85 (55.9)	172 (55.5)
	Non-Hispanic	71 (44.9)	67 (44.1)	138 (44.5)
Sexual health attitudes score, mean (SD)		10.1 (2.4)	10.1 (2.5)	10.1 (2.5)
Sexual health knowledge score, mean (SD)		6.5 (2.8)	6.1 (2.6)	6.3 (2.7)
Intentions to delay initiation of sex score, mean (SD)		14.9 (2.0)	14.6 (2.2)	14.8 (2.1)

**Table 2:** Delay of initiation of sexual intercourse by study condition.^a^

Behavior		Control		PlayForward		Total, n (%)	*P*^c^
		n (%)	95% CI^b^	n (%)	95% CI^b^		
**Baseline**							
	Delay of initiation of sexual intercourse	165 (100)		162 (100)		327 (100)^a^	
**6 Weeks**							>.99
	Delay of initiation of sexual intercourse	132 (97.8)	93.6-99.5	132 (98.5)	94.7-99.8	264 (98.1)	
	Initiation of sexual intercourse	3 (1.8)		2 (1.2)		5 (1.5)	
**3 Months**							.72
	Delay of initiation of sexual intercourse	133 (97.8)	93.7-99.54	127 (97.0)	92.4-99.2	260 (97.4)	
	Initiation of sexual intercourse	3 (1.8)		4 (2.5)		7 (2.1)	
**6 Months**							.72
	Delay of initiation of sexual intercourse	126 (97.7)	93.4-99.5	120 (96.8)	92.0-99.1	246 (97.2)	
	Initiation of sexual intercourse	3 (1.8)		4 (2.5)		7 (2.1)	
**12 Months**							>.99
	Delay of initiation of sexual intercourse	123 (95.4)	90.2-98.3	122 (94.6)	89.1-97.8	245 (95.0)	
	Initiation of sexual intercourse	6 (3.6)		7 (4.3)		13 (4.0)	

^a^A total of six participants (control: n=2; PlayForward: n=4), who at baseline were identified (per primary outcome definition) as being engaged in sexual intercourse, were removed from the primary analysis because they had already reached the primary outcome.

^b^Exact 95% confidence intervals are provided for the main outcome (delay of initiation of sexual intercourse).

^c^*P* values are based on a two-sided Fisher exact chi-square test.

## Discussion

### Principal Findings

In a cohort of community-based racial/ethnic minority adolescents, we found low rates of initiation of sexual intercourse over the 12-month follow-up in the PlayForward and control groups. Those who played PlayForward demonstrated greater improvement in attitudes around sexual health and greater increases in sexual health knowledge over 12 months than the control group.

### Interpretation

This study is unique in that it is based in community settings targeting HIV risk in teens using engaging and novel methods that possess the potential for widespread dissemination and impact. To our knowledge, this is the first randomized controlled trial demonstrating the efficacy of a portable sexual health serious video game intervention impacting sexual attitudes and knowledge. The PlayForward game includes many components of programs that have been shown to be effective in reducing sexual risk in adolescents [53,54], such as tailoring programs for the target population, using theory to guide program development, addressing more than just sexual risk, and targeting behaviors most amenable to change. PlayForward was developed with the input of the target audience allowing for its cultural appeal for this population. It incorporates social learning theory and self-efficacy [34,35] and principles from message framing [36] grounded in prospect theory [37]. It addresses myriad risk situations adolescents face, including but not limited to sex and a range of sexual risk behaviors and critical antecedents to those behaviors including attitudes, knowledge, and intentions, with the goal of impacting youth on a number of different levels. It was designed through an iterative approach that focused on our target audience and employed qualitative research methods as well as mixed methods to create and refine the final intervention [55]. Although the theories that serve as the foundation for the game focus on individual reflective processes and the assessments measured these specific processes, the intervention does not disregard automatic processes. Throughout the game, individuals practice their decision-making skills within social contexts. In the narrative sections of the game, the players are challenged to explore the risk around them and learn how that risk influences their individual processes. Where the theory does not specifically target automatic processes, the gameplay does address these processes. Although the game is comprised of up to 16 hours of unique gameplay content, the goal was to expose players to 8 to 12 hours of the intervention given that this amount of time is consistent with existing HIV prevention interventions for adolescents. Athough the median amount of gameplay was 10 hours, many of the players completed the game. An earlier analysis examined the important question as to whether there is a relationship between exposure to different intervention components and study outcomes [39]. This study revealed that it was not the duration of gameplay that seemed to be important in impacting outcomes, but the quality (ie, how well they played) of the participants’ performance in specific intervention components of the game.

Our findings are consistent with and complement findings from studies demonstrating the effectiveness of school-based interventions targeting sexual risk reduction in adolescents of similar ages and racial/ethnic backgrounds [42,56,57]. Notably, two of the studies had similar rates of sexual initiation at baseline (4-9%) [42,57] as this study, whereas the third study had considerably higher rates of initiation (23%), indicating it may have been a higher risk population [56]. Similar to this study, one study demonstrated increased positive sexual health attitudes in boys only and increased HIV-related knowledge in boys and girls [57]. This study’s intervention delayed sexual initiation and had an impact on other psychosocial determinants in boys, but had a limited effect for girls. The reason for our study’s finding of an impact on boys and not girls for sexual health attitudes is not entirely clear. One contributing factor could be that boys, compared to girls, reported feeling more connected to their character in the game (60% vs 39%, *P*=.01) and this connection could conceivably result in a greater impact on boys’ attitudes (unpublished data). Two school-based sexual risk interventions [42,56] demonstrated a delay of initiation of sex in middle-school students, although one did not show an effect in boys or in African-American students [42]. All these studies [42,56,57] required either trained facilitators and/or group discussions as part of the intervention and one study included an additional computer component [42], as compared to this study in which the video game was a stand-alone intervention. In addition, they all followed participants for 24 months, with participants being older at follow-up, allowing for a potentially greater number of events of sexual initiation.

Although there have been long-standing efforts for comprehensive sex education, many challenges for school-based programs remain. Barriers to implementation include competing priorities, lack of parental and administration support, and lack of training [58-61]. In one study, school staff reported confidence in discussing sexuality, but reported varying levels of support for comprehensive sex education from parents (42%), community leaders (53%), and school officials (50%) [60]. In contrast, our qualitative study of key stakeholders implementing PlayForward in real-world settings demonstrated support for the game intervention’s potential role in sexual health education in schools [62]. PlayForward offers potential implementation advantages over other school-based interventions because it does not involve intensive training requiring significant human and financial resources, and it provides a level of fidelity in the delivery of content. Importantly, the PlayForward intervention incorporates components of the operational guidance on comprehensive sexuality education promoted by international organizations [63]. These include the provision of scientifically accurate information, a safe learning environment, participatory teaching approaches, strengthening adolescents’ skills in communication, decision making, and critical thinking, youth advocacy, and civic engagement in program design and cultural appropriateness, tailored for distinct subpopulations. Technology-based platforms such as the PlayForward video game intervention offer unique advantages because their content and graphics can be rapidly and inexpensively modified and updated, allowing for adaptation of the intervention for different populations and outcomes [64]. Therefore, the PlayForward intervention responds to the call for rigorously evaluated interventions using technology for HIV prevention in adolescents [65]. As the use of digital health interventions is expanding and holds great promise, it is important that they are subject to serious evaluation. Our use of a randomized controlled trial is justified by the ensured stability and engagement of the delivery vehicle of our intervention and that it was delivered with documented fidelity. Given the engagement of our target population in its development and the substantial exposure of our participants to the intervention over time, it has a considerable likelihood of having a clinically meaningful impact [66].

Although we adhered to standards regarding evaluating behavioral interventions [67], some limitations of this study must be considered. A strength of this study is that the intervention was built on a solid foundation of well-established theories, but these theories apply most specifically to behavior change and may have less utility for a prevention intervention where the goal is for the behavior to remain the same. There is considerable precedent in the literature for grounding prevention interventions within a behavior change framework; however, in doing this we may not be accounting for factors that may be relevant to prevention but not behavior change. Notably, the low rate of initiation of sexual intercourse precluded us from determining PlayForward’s impact on the primary outcome at 12 months. These low rates are consistent with current national figures [68] with 4% of high school students reporting sexual intercourse before age 13, increasing to 24% by ninth grade (approximately 15 years). Therefore, 12-month follow-up for our cohort may be too short to capture these events. Our outcomes were based on self-report, which can introduce biases in terms of disclosure of sensitive information. As described elsewhere [26], we used data collection methods that optimized disclosure and the accuracy of self-reported data, and ensured privacy and confidentiality in this age group [69,70]. We chose to use face-to-face assessments as we were collecting large amounts of data (15 different assessment instruments) in a young teen cohort and wanted to ensure completion of the assessments. We were cognizant of issues around disclosure given that some of the assessments included sensitive data (sex- and substance use-related data). There are differing views in the literature examining paper versus computer-based assessments of sensitive data [69], but research has found that paper versions elicited higher and more accurate rates of disclosure [71], demonstrated more skipped items, and had no specific advantage to Web-based interviews [70]. Although we studied a population of racial/ethnic minority adolescents who, as a group, are at increased risk for HIV/STIs, they were involved in structured programs and, therefore, the findings may not translate to other populations including those not engaged in afterschool or school-based programs [72].

Despite these limitations and the low event rate of sexual initiation in our cohort, we demonstrated a compelling and persistent impact on attitudes and knowledge. There is evidence for a correlation between attitudes and behavior [73-75], especially in the case of strong attitudes [76]. One study examining survey data from over 1700 ninth graders analyzed the relationship between a number of variables including attitudes and specific outcomes, one being the behavior of abstaining from sexual intercourse [75]. This study found that attitudes had the strongest relationship, not only with intentions (beta=.48) but, more importantly, with behavior (beta=–.34). This finding demonstrated a relationship with students who had high scores indicating more conservative attitudes about having sex being less likely to have sex (*P*=.01). Furthermore, one meta-analysis concluded that changing attitudes could produce a significant impact specifically on stemming the HIV epidemic [77]. In addition to digital health interventions such as video games being effective, there remains a critical need for those interventions targeting sexual health to be accessible, adaptable, easily disseminated, and delivered with fidelity. Given the growing focus on the use of digital health (including sexual health [78]) to increase access to and engagement with interventions, this study provides evidence for this approach. According to the 2016 National Education Technology Plan, “technology increasingly is being used to personalize learning and give students more choice over what and how they learn and at what pace, preparing them to organize and direct their own learning for the rest of their lives” and the focus is on “using technology to transform learning experiences with the goal of providing greater equity and accessibility” [79].

### Conclusions

Serious video games as digital health interventions offer the unique opportunity to increase the accessibility and reach of theory-driven and tested interventions. The PlayForward intervention demonstrated efficacy in improving sexual attitudes and knowledge in racial/ethnic minority adolescents that persisted up to 12 months. Future research should assess PlayForward’s efficacy in populations with higher rates of sexual initiation and its comparative efficacy, effectiveness, and implementation.

## Appendix

Multimedia Appendix 1 PlayForward homescreen.

Multimedia Appendix 2 A Challenge Stack: 7 Minutes in Heaven.

Multimedia Appendix 3 Know Sense mini-game.

Multimedia Appendix 4 People Sense mini-game.

Multimedia Appendix 5 CONSORT-EHEALTH checklist (v1.6.1).

## Abbreviations

AIDS: acquired immune deficiency syndrome
DSMB: data and safety monitoring board
HIV: human immunodeficiency virus
LS: least squares
STI: sexually transmitted infection
