# Supplementary figures and images for: Video Game Intervention for Sexual Risk Reduction in Minority Adolescents: Randomized Controlled Trial
Source: J Med Internet Res. 2017 Sep 18;19(9):e314. doi: 10.2196/jmir.8148 (PMC5625130; doi:10.2196/jmir.8148)

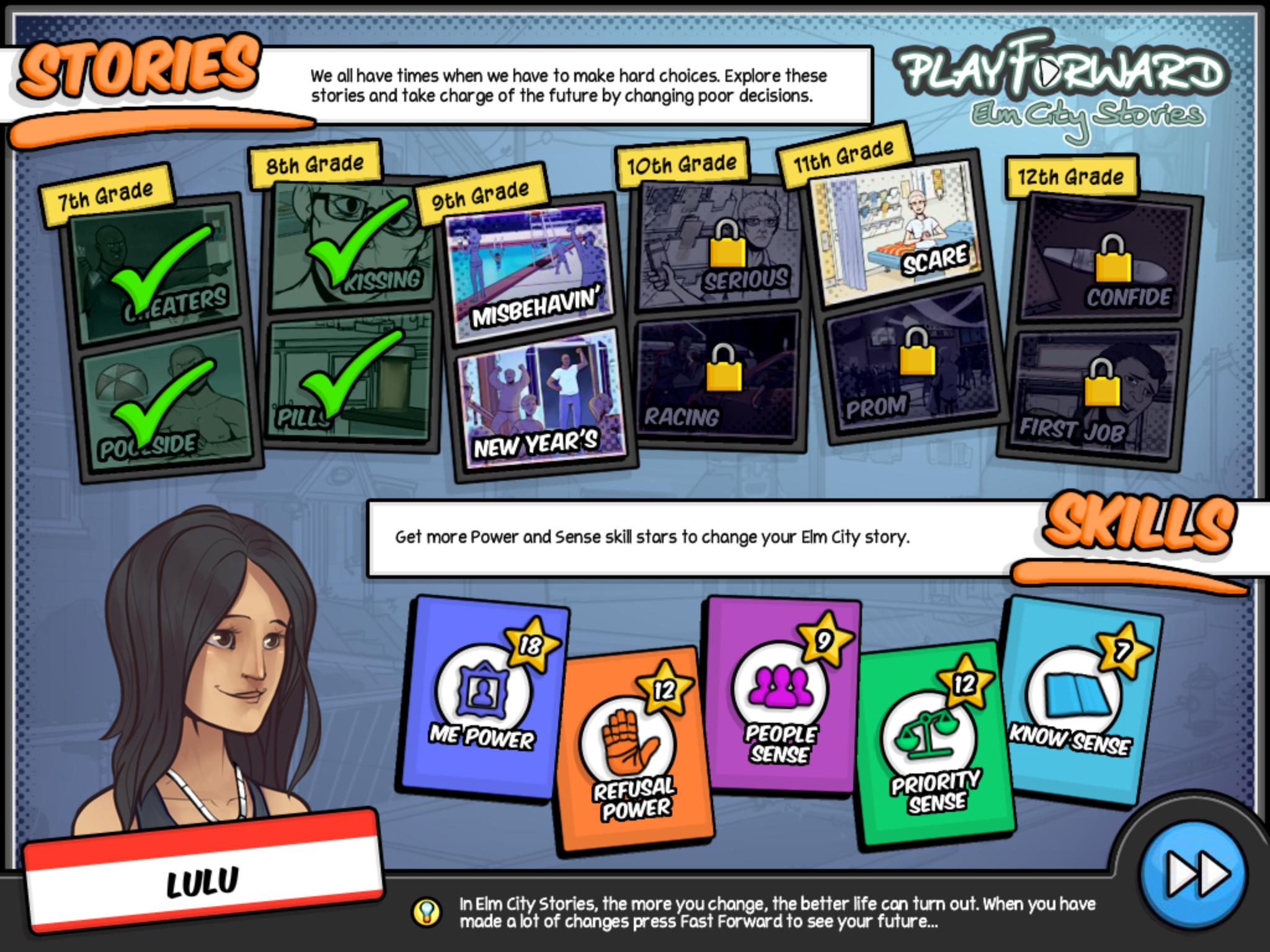

Supplement: Multimedia Appendix 1 [file jmir_v19i9e314_app1.PNG]

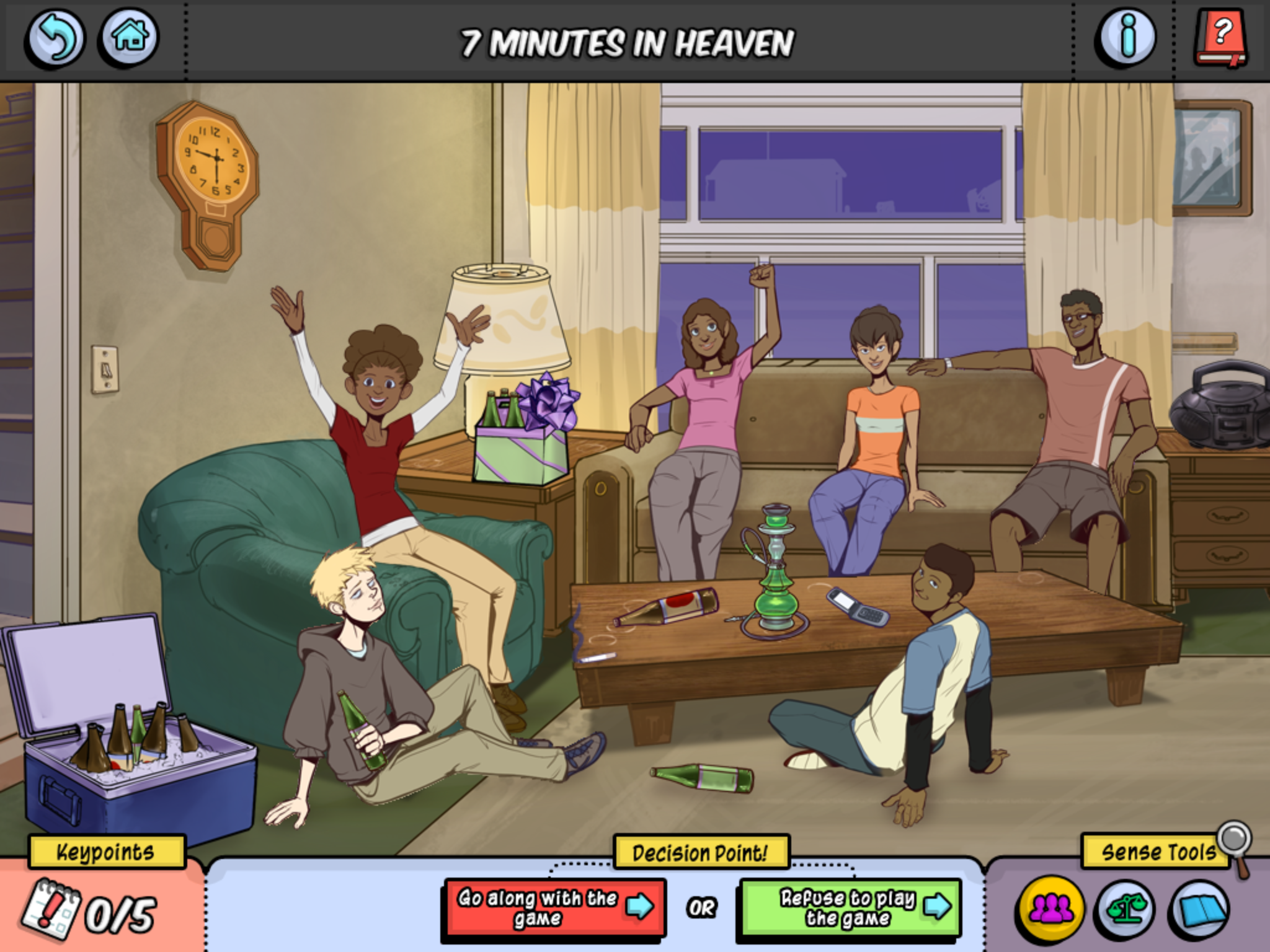

Supplement: Multimedia Appendix 2 [file jmir_v19i9e314_app2.PNG]

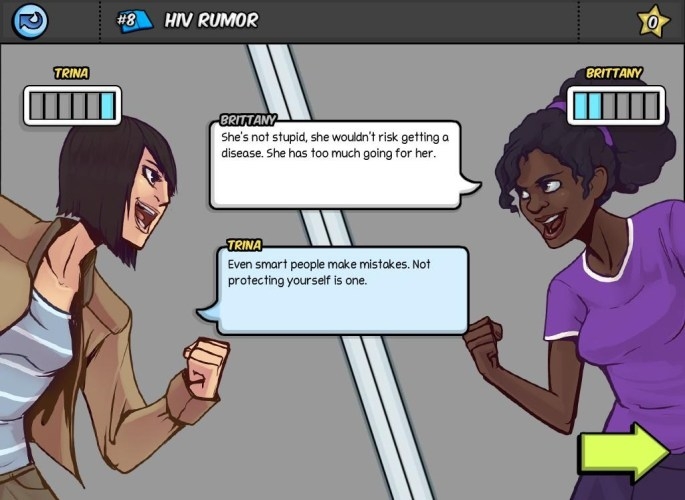

Supplement: Multimedia Appendix 3 [file jmir_v19i9e314_app3.JPG]

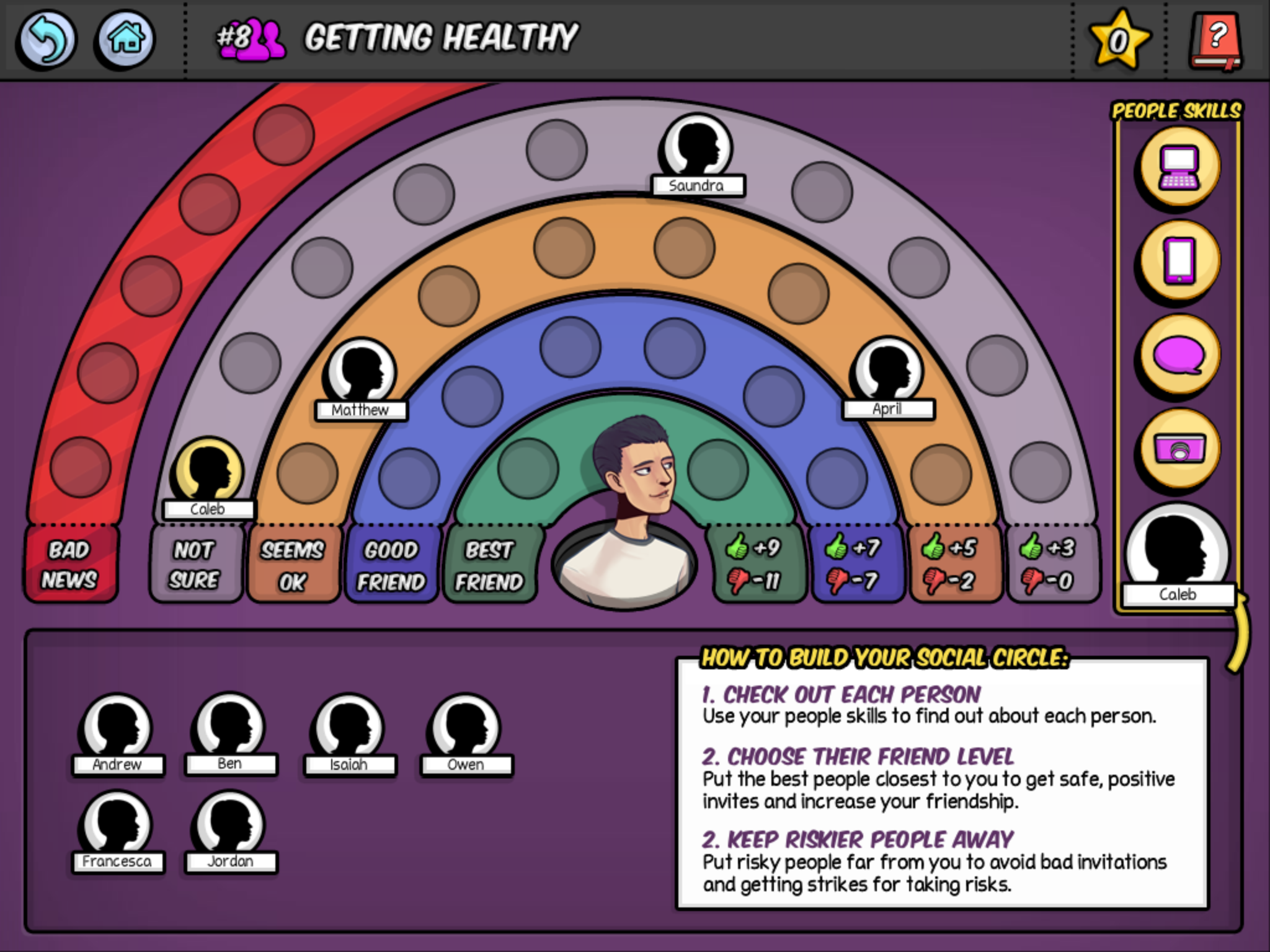

Supplement: Multimedia Appendix 4 [file jmir_v19i9e314_app4.PNG]
